# Supplementary material for: Alternative Approach for Specific Tyrosinase Inhibitor Screening: Uncompetitive Inhibition of Tyrosinase by Moringa oleifera
Source: Molecules. 2021 Jul 29;26(15):4576. doi: 10.3390/molecules26154576 (PMC8348646; doi:10.3390/molecules26154576)
Supplement: Supplementary file 1 [file molecules-26-04576-s001.zip › molecules-1264850-supplementary.pdf]

Supplementary Material

Article

Alternative Approach for Specific Tyrosinase Inhibitor Screening: Uncompetitive Inhibition of Tyrosinase by *Moringa Oleifera*

Farah J. Hashim [1,2,3], Sukanda Vichitphan [2,4], Jaehong Han [5,*] and Kanit Vichitphan [2,4,*]

[1] Graduate School, Khon Kaen University, Khon Kaen 40002, Thailand; farah_bio_2020@yahoo.com
[2] Department of Biotechnology, Faculty of Technology, Khon Kaen University, Khon Kaen 40002, Thailand; sukanda@kku.ac.th
[3] Department of Biology, College of Science, University of Baghdad, Baghdad 10071, Iraq
[4] Fermentation Research Center for Value Added Agricultural Products (FerVAAP), Khon Kaen University, Khon Kaen 40002, Thailand
[5] Metalloenzyme Research Group and Department of Plant Science and Technology, Chung-Ang University, Anseong 17546, Republic of Korea
* Correspondence: jaehongh@cau.ac.kr (J.H.); kanvic@kku.ac.th (K.V.)

Table S1. TYR diphenolase inhibition (%) by luteolin and *Moringa oleifera* extract in the presence and absence of triton X-100.

	**Triton X-100**			
	0	0.01%	0.05%	0.10%
**Luteolin, 35 μg/ml**	50.9 (±1.4)	49.1 (±2.3)	50.7 (±0.2)	49.6 (±2.4)
***M. oleifera*, 75 μg/ml**	51.4 (±2.4)	49.2 (±1.9)	51.1 (±2.5)	49.8 (±2.5)

Table S2. Identification of compounds in the extract of *Moringa oleifera* leaves by HPLC/MS analysis.

Peak at **Figure 6**	Retention time (min)	$[M-H]^-$ (*m/z*)	Expected MF from MS library	Tentative identification
**a**	1.96	357.1032	$C_{12}H_{22}O_{12}$	Lactobionic acid
**b**	3.13	337.0764	$C_{12}H_{18}O_{11}$	L-Ascorbic acid-2-glucoside
**c**	37.04	651.1553	$C_{29}H_{32}O_{17}$	Luteolin 7-(6'''-acetylallosyl-(1→2)-glucoside)
**d**	42.90	475.0873	$C_{22}H_{20}O_{12}$	Luteolin 7-methylglucuronide
**e**	51.03	425.1924	$C_{20}H_{30}N_2O_8$	3-Hydroxylidocaine glucuronide
**f**	80.83	285.0401	$C_{15}H_{10}O_6$	Luteolin

Figure S1. Mass spectrum of six components of *Moringa oleifera* leaf extract, (a) luteolin; (b) Luteolin 7-methyl glucuronide; (c) Luteolin 7-(6'''-acetylallosyl-(1→2)-glucoside); (d)

lactobionic acid; (e) 3-hydroxylidocaine glucuronide; (f) L-ascorbic acid-2-glucoside; (g) melanostatin.

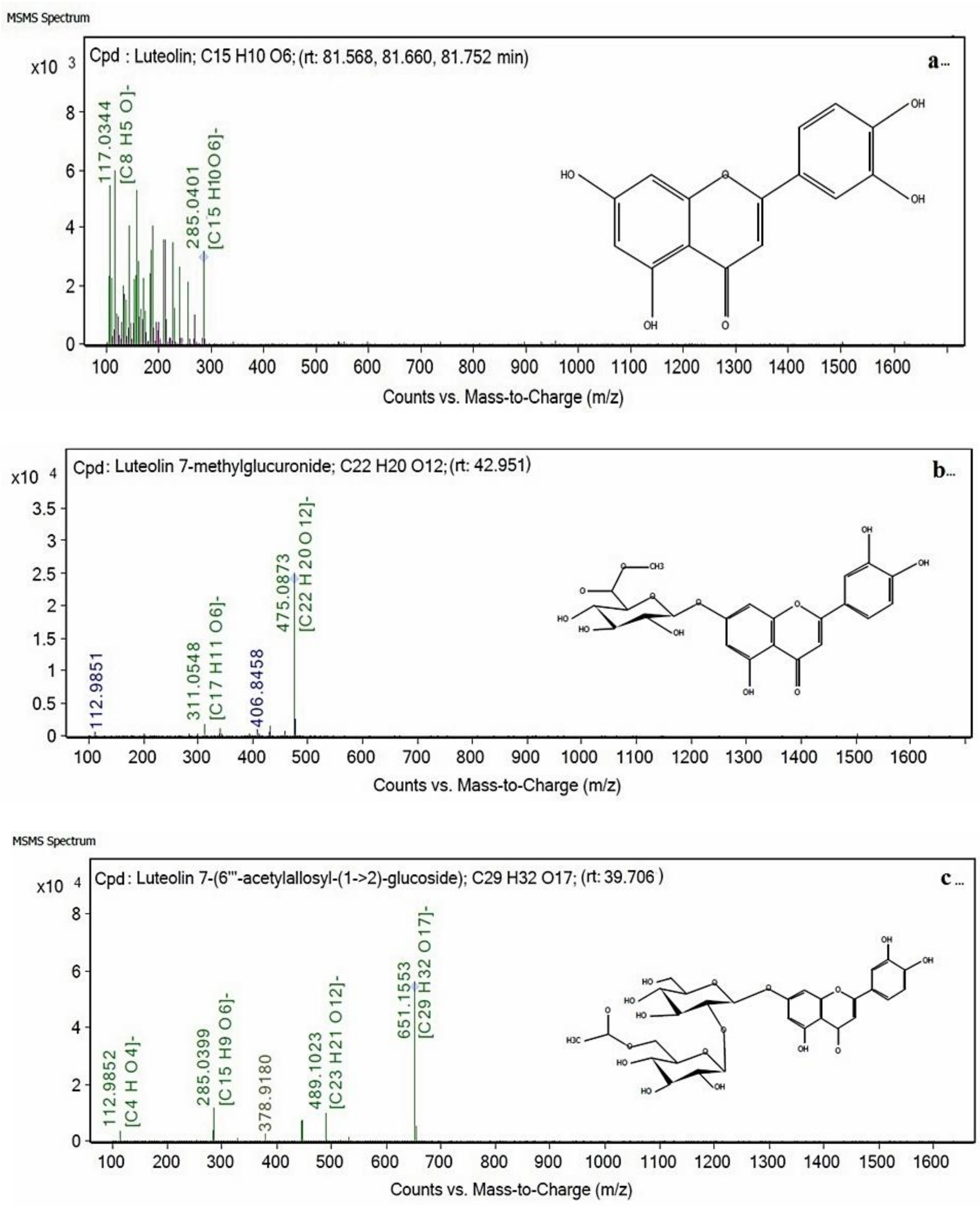

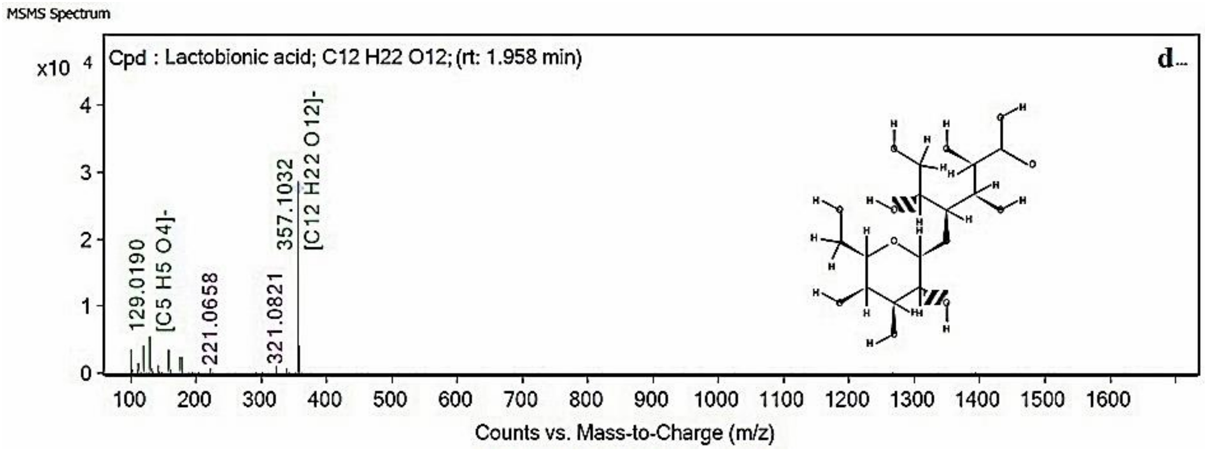
MSMS Spectrum
Cpd : Lactobionic acid; C12 H22 O12; (rt: 1.958 min)
d...
x10 4
129.0190
[C5 H5 O4]-
221.0658
321.0821
357.1032
[C12 H22 O12]-
Counts vs. Mass-to-Charge (m/z)

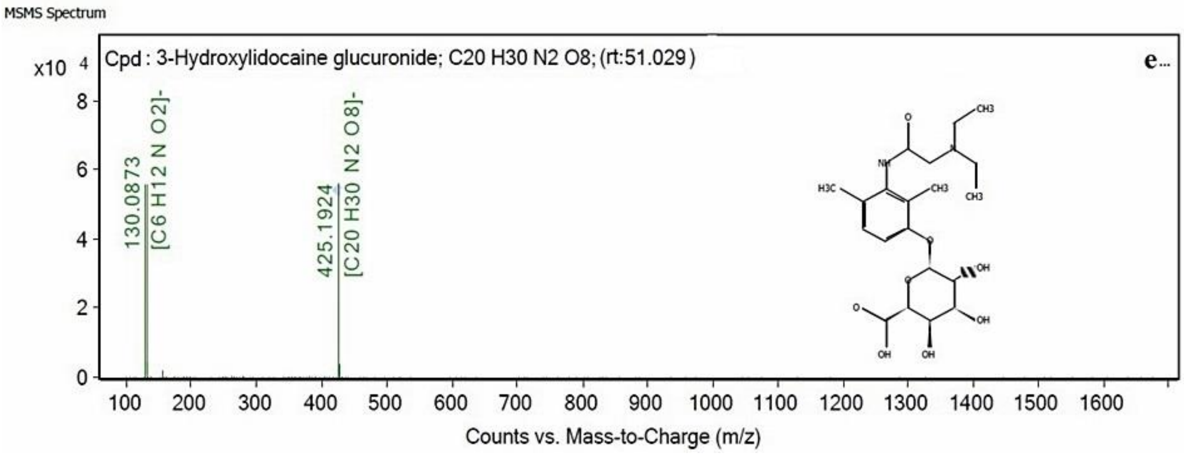
MSMS Spectrum
Cpd : 3-Hydroxylidocaine glucuronide; C20 H30 N2 O8; (rt:51.029)
e...
x10 4
130.0873
[C6 H12 N O2]-
425.1924
[C20 H30 N2 O8]-
Counts vs. Mass-to-Charge (m/z)

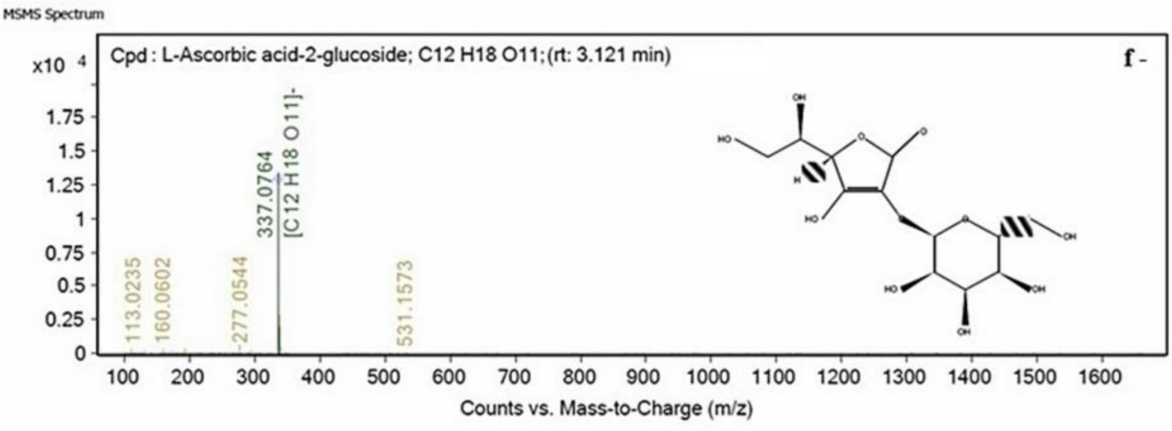
MSMS Spectrum
Cpd : L-Ascorbic acid-2-glucoside; C12 H18 O11; (rt: 3.121 min)
f -
x10 4
113.0235
160.0602
277.0544
337.0764
[C12 H18 O11]-
531.1573
Counts vs. Mass-to-Charge (m/z)

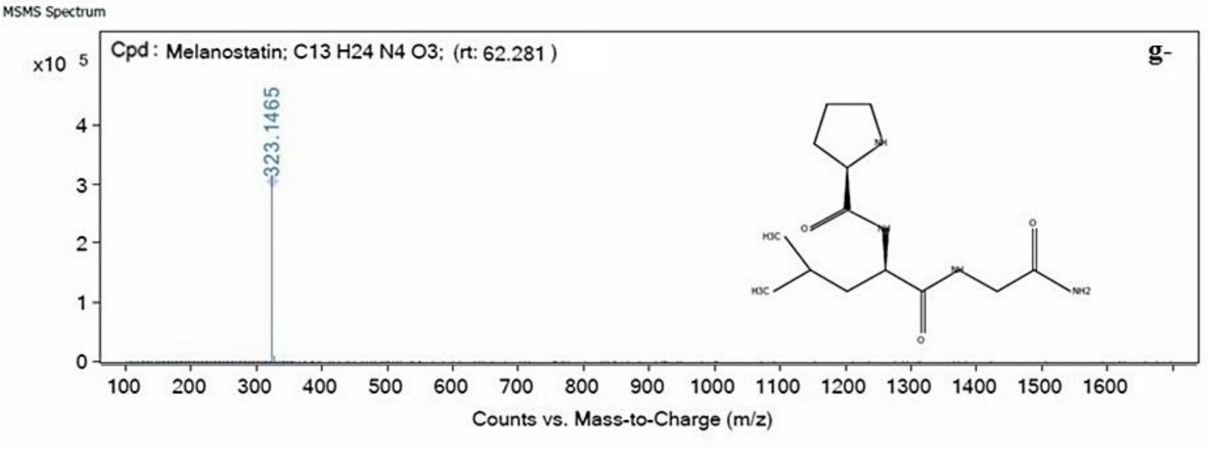

Luteolin quantification by HPLC

- **Sample Preparation and Standard Solutions**

Moringa extract (1 g) was dissolved in 50% methanol (1 ml). The sample was filtered to remove undissolved particles. Subsequently, 20 µl was injected in the instrument. Meanwhile, reference compound (luteolin) was dissolved in 50% methanol to obtain standard curve with final conc. (5, 10, 20 and 40 µg/ml).

- **Chromatography conditions and analysis**

A HPLC (LC-10D, Shimadzu, Kyoto, Japan) equipped with a UV detector (Shimadzu, Kyoto, Japan) and Waters-C18 column (4.6 mm × 75 mm; 3.5 µm particle size) was used to separate the compounds. The mobile phase consisted of (A) 0.1% formic acid in water and (B) acetonitrile , with the following gradient elution program: 98% A, 0- 2 min; 90% A, 2- 25 min; 85% A, 25-40 min; 80% A, 40-48 min; 75% A, 48-68 min; 70% A, 68-80 min; 50% A, 80-85 min; 0% A, 85- 90 min.; 98% A, 90-100 min. Peaks were detected at the wavelengths of 254 nm. The quantification of luteolin compound was calculated based on a standard curve of luteolin pure compound 10 µl injection volume of standard and sample solutions was used.

Results

Data of HPLC (Figure 1S) revealed that luteolin conc. about 15.9 mg/g of moringa extract (~1.6%) which is significantly higher than luteolin content in moringa sample (~94 µg/gm of moringa extract) obtained from Mexico [1]. The reason behind high conc. of luteolin phytochemical constituents of our sample might be due to effect of environmental conditions on the plant including the climatic environment, soil factors such as soil type, pH, and soil nutrients [2]. Therefore, Concentrations of these active principles usually associated with influences such as environment and growing regions [3].

Figure S2. HPLC chromatogram of (a) Luteolin pure compound, (b) *Moringa oleifera* leaves extract.

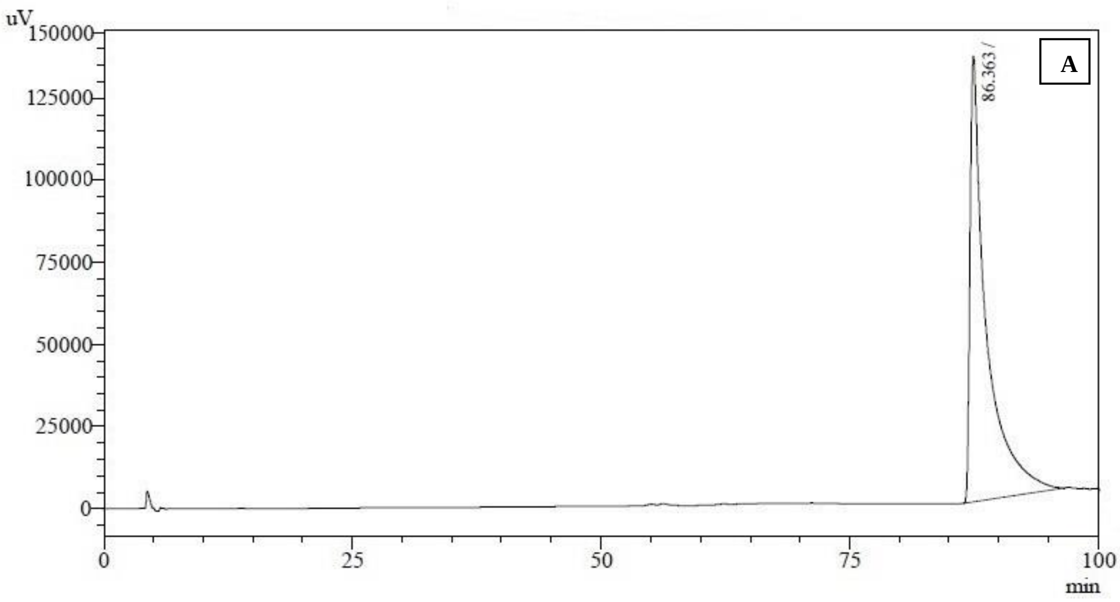

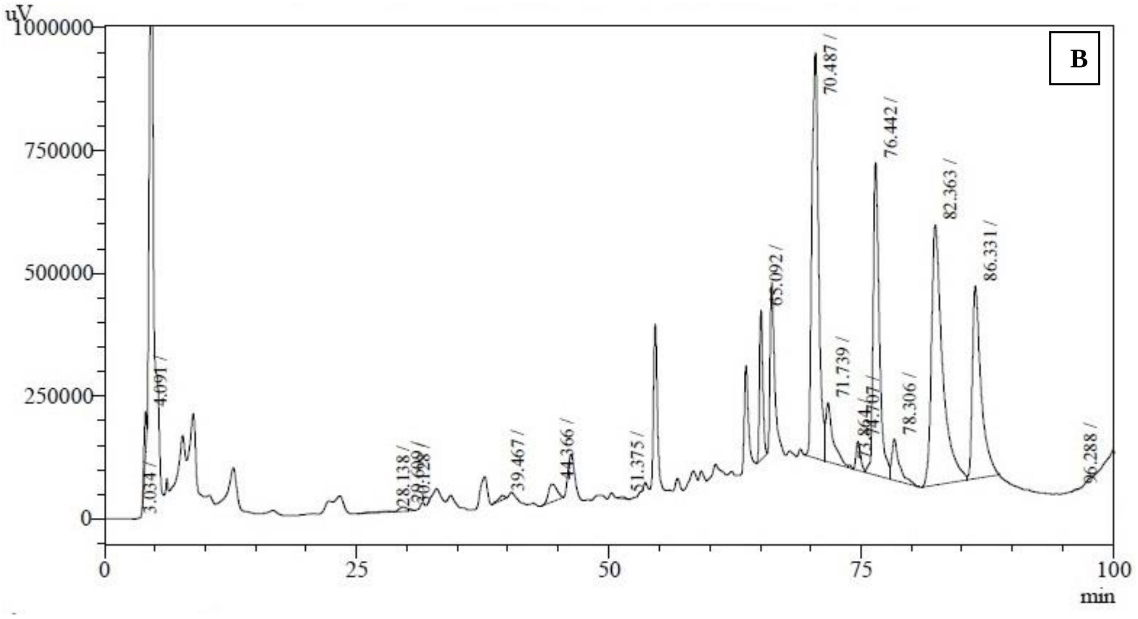

References

1. Valdez-Solana MA, Mejía-García VY, Téllez-Valencia A, García-Arenas G, Salas-Pacheco J, Alba-Romero JJ, Sierra-Campos E (2015) Nutritional Content and Elemental and Phytochemical Analyses of Moringa oleifera Grown in Mexico. journal of chemistry 1-9.
2. Wright RJ, Lee KS, Hyacinth HI, Hibbert JM, Reid ME, Wheatley AO, Asemota HN (2017) An investigation of the antioxidant capacity in extracts from Moringa oleifera plants grown in Jamaica. Plants 6(48): pp. 2–9.
3. Sulastri E , Zubair MS, Anas NI , Abidin S , Hardani R , Yulianti R , Aliyah (2018) Total phenolic, total flavonoid, quercetin content and antioxidant activity of standardized extract of Moringa oleifera leaf from regions with different elevation. Pharmacognosy Journal. 10(6): s104–s108.
